# Everybody's Page

**Published:** 1892-11-12

**Authors:** 


### Everybody's Page.

According to an American contemporary, a physician prescribes change to a patient and then takes all he has.

Acting, no doubt, on the belief that flies can be the means of disseminating various diseases, the police authorities of a certain town in Prussia are alleged to have placed their ban upon flies as contributories to the spread of cholera. All good citizens were, therefore, warned to spare neither time nor energy in pursuit even to death of every fly in their houses. Their advice might with advantage be followed out in other than cholera times, for flies are never anything but objectionable companions.

Attention has been called officially in Marylebone to the practice which holds widely in that parish of placing servants to sleep in cellars and basements which are unfit for human habitation. It is an open secret that Marylebone is not the only parish in which this reprehensible custom is practised, and that some of the greatest offenders in this direction are wealthy householders who display a carelessness as to the comfort and sanitary surroundings of their servants for which the application of the epithet reprehensible is mild.

Among the numerous exhibits which will, no doubt, attract attention at the Chicago World's Fair is to be one of anthropology. A strip of land along a lagoon will be occupied by groups of native Americans, who will be visible in their natural habitations. In addition to a representative exhibit of the remains of historic man in America, there will be large models of the most remarkable of their works, and an exhibition of primitive industries and customs. Such an exhibit has possibilities of being made extremely interesting and attractive.

Foreign physicians are agreed almost unanimously as to the importance of adopting immediately the repressive treatment of cholera by opium, acting upon the theory which assumes the worst symptoms of cholera to be the result of loss of fluid. Authorities in this country though divided are now for the most part directly opposed to this view, which they maintain to be inconsistent with the acknowledged fact of the disease. If it is correct that the symptoms of choleraic collapse are fully accounted for by the impeded circulation through the lungs, the treatment of cholera by mild laxatives would appear to be the right one.

The use, or perhaps more correctly, the abuse of opium is the subject of frequent controversy and attack, but hasheesh, prepared from the grains of Egyptian hemp, and used both in a liquid form as drink and as a tobacco, has not come in for the same amount of vituperation. Its abuse is probably less to be condemned, though it inevitaly brutalizes those who make a continued use of it, and they are many. As an excitant of the brain it imparts a shock to the nervous system. Those who have studied its action state that victims of a craving for hasheesh, in contradistinction to consumers of opium, are both active and passive. The hasheesh eater begins by struggling against ideas which he knows to be false with the energy of one who feels that his reason is escaping from him. As a writer in the Paris *Revue des Revues* states, such a man is wide awake and has no desire to sleep. Time and space and past and future have no existence for him, and nothing exists for him outside of the phenomena produced within him. Those who are in search of a new crusade will find a field for their energies in attacking this pernicious habit.

